# Midgut immune profiling and functional characterization of *Aedes aegypti* ABC transporter gene(s) using systemic and local bacterial challenges

**DOI:** 10.1186/s13071-025-06658-6

**Published:** 2025-01-31

**Authors:** Vikas Kumar, Shilpi Garg, Diksha Sisodia, Lalita Gupta, Sanjeev Kumar, Vishal Saxena

**Affiliations:** 1 Department of Biological Sciences, Birla Institute of Technology and Science (BITS), Pilani Campus, Pilani, 333031 Rajasthan India; 2 Department of Zoology, Chaudhary Bansi Lal University, Bhiwani, Haryana India; 3 Department of Biotechnology, Chaudhary Bansi Lal University, Bhiwani, Haryana India

**Keywords:** *Aedes aegypti*, ABC transporters, Midgut Immunity, Bacteria, RNA interference

## Abstract

**Background:**

The mosquito midgut is crucial for digestion and immune interactions. It produces several immune factors that protect the organ from invading pathogens and can limit their propagation. Studies on mosquito midgut transcriptome following pathogen exposure have revealed the presence of non-canonical immune genes, such as ABC transporters, whose function in insect immunity remains unexplored. Therefore, this study focuses on identifying and characterising the immune role of ABC transporters in the midgut of *Aedes aegypti*, a primary arboviral vector.

**Methods:**

To identify the midgut-expressed ABC transporters, the mosquitoes were challenged with a mixture of gram-negative (*Escherichia coli*) and gram-positive (*Micrococcus luteus*) bacteria, and the expression of all ABC transporters was analysed with PCR using gene-specific primers. Furthermore, the transcriptional alterations of midgut ABC transporters were explored at different time points upon a thoracic nano-injection (systemic challenge) or infectious blood meal (local challenge) of the bacterial mixture through quantitative real-time PCR (qPCR), and one gene was selected for RNAi-mediated gene silencing and its role assessment in midgut immune responses.

**Results:**

The expression of all 48 microbial-induced midgut-expressing *Ae. aegypti* ABC transporter genes upon systemic or local bacterial challenges was analyzed. Based on the transcriptomic data and potential immune expression similar to the well-known immune gene *defensin, AaeABCG3* was selected for RNAi-mediated gene silencing and characterization. The *AaeABCG3* gene silencing exhibited a significant reduction of midgut bacterial load through the induction of nitric oxide synthase (*NOS*) in sugar-fed and systemic bacterial-challenged mosquitoes. In contrast, midgut bacterial load was significantly regulated by induction of *defensin A* and *cecropin G* in the late hours of local bacterial challenges in *AaeABCG3*-silenced mosquitoes.

**Conclusions:**

The silencing of *AaeABCG3* modulated the mosquito midgut immune response and disturbed the midgut microbiota homeostasis. The systemic immune responses of *AaeABCG3*-silenced mosquitoes were influenced by the JAK-STAT pathway with no induction of Toll and IMD immune pathways. Interestingly, Toll and IMD immune pathways actively participated in the late hours of local bacterial challenges, suggesting that the route of infection influences these immune responses; however, the molecular mechanism behind these phenomena still needs to be explored. Overall, this work provides significant insight into the importance of ABC transporters in mosquito immunity.

**Graphical Abstract:**

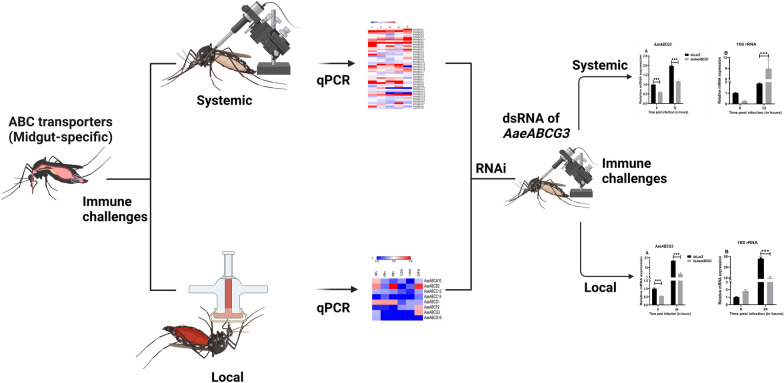

**Supplementary Information:**

The online version contains supplementary material available at 10.1186/s13071-025-06658-6.

## Background

Mosquitoes constantly face infection with various pathogens in their natural habitat and encounter several microorganisms during the aquatic developmental stages. Adults can be exposed to different microbes through nectar feeding and physical injuries. In addition, their haematophagous nature exposes them to microbes of the host skin and different blood-borne pathogens like dengue virus (DENV), Zika virus (ZIKV), and malaria parasite *Plasmodium* through blood-feeding [1, 2]. The mosquito midgut is the first organ to encounter and mount an array of immune responses against an ingested pathogen. In addition to being the organ of digestion, the mosquito midgut is also home to various naturally acquired microbiota required for digestion and nutrition [3, 4]. It is also a unique site for tripartite interactions among midgut microbiota, ingested pathogens, and mosquito innate immune systems. These reciprocal interactions could affect vector physiology and significantly impact immune responses [5, 6]. Therefore, the mosquito midgut is considered an organ of interest in controlling vector-borne diseases.

The immune signalling pathways like the Toll, immune deficiency (IMD), and Janus kinase signal transducer and activator of transcription (JAK-STAT) pathways are central to mosquito midgut innate immune response against invading pathogens [1, 7]. The Toll pathway is regulated by transcription factor Rel1 and tends to be induced against gram-positive bacteria, fungi, and viruses [1, 8–10], where it activates cellular immunity and increases the expression of the bactericidal peptides [9, 11, 12]. Its role against the dengue virus (DENV) has also been reported in *Aedes aegypti* [10]*.* The IMD pathway responses are controlled by the transcription factor Rel2 [1, 7]. This pathway is essential for controlling gram-negative and some gram-positive bacterial infections through the production of antimicrobial peptides (AMPs), where each AMP gene is reported to be expressed with different kinetics, suggesting that the transcription of AMP genes is a complex process [12–15].

The Toll and IMD pathways have overlapping molecules for eliciting an immune response. Interestingly, these pathways could co-regulate several AMPs, like *defensins* and *cecropins. Defensins* are the most effective antimicrobial peptides against gram-positive bacteria [7, 16, 17], while c*ecropins* are predominantly responsive against gram-negative bacterial infection [18]. The reactive oxygen/nitrogen species (ROS/RNS) generation by dual oxidase enzyme (*DUOX*, an NADPH oxidase family member) or nitric oxide synthase (*NOS,* a downstream effector molecule of the JAK/STAT pathway) genes plays a crucial role in modulating the indigenous microbiota proliferation, opportunistic pathogenic bacterial growth, and arbovirual infection in the mosquito midgut [4, 19–21].

Recent studies in *Anopheles stephensi*, where mosquitoes were infected through bacterial feeding and bacterial microinjection, showed that tissues could synergistically cope with a local (endogenous) and systemic (exogenous) infection after microbial exposure, respectively [22]. The local immune defence largely relies on antimicrobial peptides (AMPs) produced by the immune signalling pathways and the physical barrier [6, 22, 23]. Bacterial challenges through thoracic injury cause induction of *NOS* in insect haemolymph, indicating the importance of ROS/RNS in systemic bacterial immune responses of the *Anopheles gambiae* mosquito [1, 24]. Recently, a transcriptional alteration of putative immune genes in the salivary gland of *An. coluzzii* was also reported upon local and systemic immune challenges [25]. The local and systemic immune responses and molecular communications between various organs/tissues have been well documented in insects like *Drosophila* and mosquitoes [22, 26].

Interestingly, some studies have shown that non-canonical immune molecules like ABC transporters exhibit transcriptomic alteration against microbial infections in mosquitoes [27]. The role of ABC transporters in insect immunity has also been reported in the fly and dengue virus vector in *Ae. aegypti* [28, 29], where they are commonly known for their role in insecticide or multidrug resistance. They contribute to insecticide detoxification by effluxing the intracellular concentration of toxic compounds or their metabolic products [28, 30–32]. In general, ABC transporters are the transmembrane molecular channels that utilise ATP hydrolysis energy to transport various molecules across the lipid bilayer. Typically, they consist of highly conserved nucleotide-binding domains (NBDs), which are located at the cytoplasmic side of the cell membrane, and less conserved transmembrane domains (TMDs) embedded into the lipid bilayer [33]. NBDs, also known as ATP-binding cassettes, contain various conserved signature motifs like Walker A (GXXGXGK(S/T)), Walker B (φφφφD, here φ denotes a hydrophobic residue), Q-loop, D-loop, and H-motif, as well as C-motif (LSGGQ), and hydrolyse ATP for transportation of various substrates. The transmembrane domains (TMDs) form the translocation pathway and provide specificity to a substrate [34, 35].

In this study, we have identified ABC transporter(s) expressed in the midgut upon bacterial challenges from *Ae. aegypti* for their immune characterisation. For this, we have used the catalogue of all 59 *Ae. aegypti* ABC transporter genes identified in our previous study [28]. Microbe-induced ABC transporters expressed in the midgut were identified post-systemic (thoracic injection of the mixture of gram-positive and -negative bacteria) and local (oral supplement of gram-positive and -negative bacterial mix) bacterial challenges using semiquantitative PCR with gene-specific primers. Furthermore, transcriptional alteration of the ABC transporter genes was examined using quantitative real-time PCR (qPCR) upon both systemic and local immune challenge at various time points. Based on the expression observed during the immune challenges and our previous report [28] where we observed downregulaion of *AaeABCG3* in three arboviral infections, dengue, West Nile and yellow fever, ABC transporter *AaeABCG3* gene was selected for further functional characterization using RNA interference (RNAi). *AaeABCG3* gene silencing was observed to modulate the mosquito midgut immune responses, affecting the midgut microbiota homeostasis that regulate pathogen development in mosquitoes. Thus, the immune characterisation of the non-canonical molecules such as ABC transporters might offer new understandings to disrupt the transmission cycle of blood-borne pathogens [32].

## Methods

### Rearing of *Ae. aegypti* mosquitoes

Female *Ae. aegypti* mosquitoes (field-collected, molecular identified, and laboratory-reared mosquitoes from the Pilani region, Rajasthan, India) were reared in a semi-natural insectarium at 27 ± 1 °C, 80% relative humidity, and 12 h light/dark cycle as described before [28, 36, 37]. Larvae were fed on fish food (Gold Tokyo, Ahmedabad, India), while the adult mosquitoes were regularly fed on 10% sucrose solution ad libitum. Blood feeding was performed for colony propagation by allowing 4- to 5-day-old female mosquitoes to feed on anaesthetised mice. The mice were obtained from the Central Animal Facility of the Institute, and all the procedures were approved by the Institute Animal Ethics (IAEC/RES/28/7) and Institute Biosafety (BITS/IBSC/ 2018/02) Committees.

### Designing of *Ae. aegypti* ABC transporter gene(s)-specific primers

cDNA sequences of all 59 *Ae. aegypti* ABC transporter genes (identified in our previous study [28]) were retrieved from VectorBase and subjected to BLASTn search against *Ae. aegypti* genome assembly 5 (AaegL5) to identify the unique regions for an individual ABC transporter. Primers were designed from these unique regions for individual ABC transporter genes using an online primer designing tool, primer3 (https://primer3.ut.ee/). During our in silico analysis, we observed an extensive duplication of some ABC transporter genes or gene segments like *AaeABCA2* (*AaeABCA2.1*, *AaeABCA2.2*), *AaeABCA7* (*AaeABCA7.1*, *AaeABCA7.2*) and *AaeABCC4* (*AaeABCC4.1*, *AaeABCC4.2*) for which we designed common primers [28]. All primers were commercially synthesised from Integrated DNA Technologies (IDT), USA. Primers were also synthesised for *Ae. aegypti* transcription factor genes Rel1 (AAEL007696) and Rel2 (AAEL007624); effector genes *defensin A* (AAEL003841), *cecropin G (*AAEL015515), nitric oxide synthase (NOS) (AAEL009745), and dual oxidase enzyme (DUOX, AAEL007563); and housekeeping gene ribosomal protein S6 (RPS6) (AAEL000032). Primer sequences for all genes used in this study are provided in Supplementary Table 1.

### Systemic and local immune challenges to *Ae. aegypti* mosquito

We obtained gram-negative *Escherichia coli* (MTCC, no. 40) and gram-positive *Micrococcus luteus* (MTCC no. 106) bacteria from Microbial Type Culture Collection, Institute of Microbial Technology (IMTECH) Chandigarh, India. The above two bacterial strains were grown individually in LB media broth following standard protocols [38] to an A_600_ = 0.5. A 1.5-ml aliquot from each culture was mixed and centrifuged for 5 min at 5000 rpm. The pellet was washed twice with 1× Ashburner’s PBS pH 7.2 (3 mM sodium chloride, 7 mM disodium hydrogen phosphate, and 3 mM sodium dihydrogen phosphate) and resuspended in 1× Ashburner’s PBS.

**Fig. 1:**
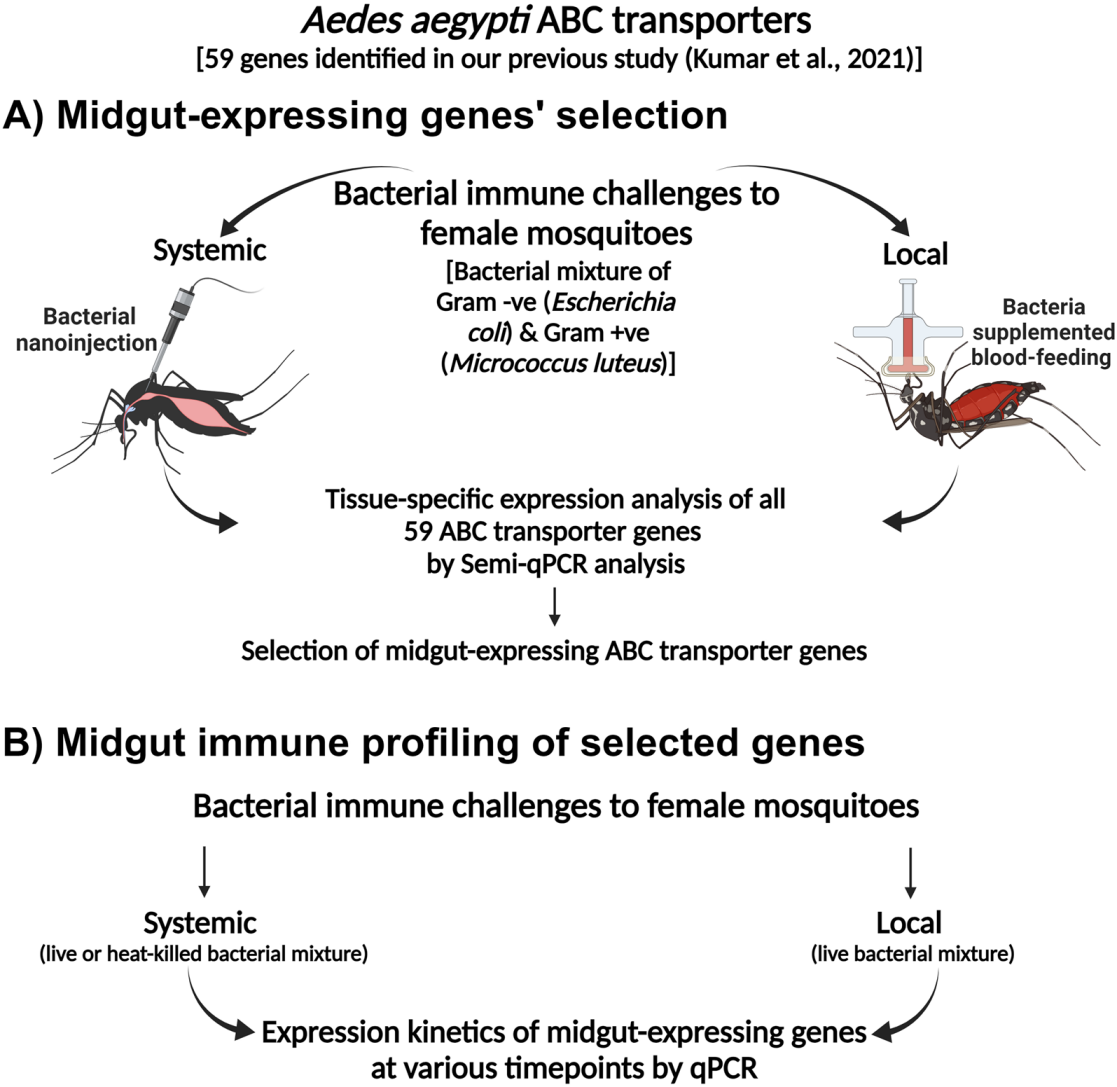
Schematics summary of mosquito immune challenge and profiling: Schematic representation for identification and selection of midgut-expressing *Aedes aegypti* ABC transporter genes through semi-quantitative PCR and qPCR upon bacterial challenge. This figure was created with BioRender.com

For systemic infection, the above mixture of bacteria (live or heat-killed) was injected into the haemocoel of mosquitoes using Nanojector injection system (Drummond, Broomall, PA, USA), as detailed before [38, 39]. The heat-killed bacteria (immune elicitors) were prepared by boiling the bacterial mixture for 5 min, as described before [38], and administered similarly to the live bacterial challenge. Uninjected and sterile PBS-injected mosquitoes served as a control in the experiment. Local infection was introduced to *Ae. aegypti* adult female mosquitoes by allowing them to feed on mousee blood supplemented with or without bacterial suspension through an artificial membrane feeder. For this, the bacterial preparation was resuspended in 3 ml heparinised mouse blood to make the final concentration of 10^9^cells/ml (finally 0.5 × 10^9^ cells of each bacterium per ml blood) as before [4, 38]. After the bacterial challenge, the midgut and carcass tissues were collected from control and infected females at different time points. Uninfected midguts and carcasses served as controls. The schematics of the experimental setup are shown in Fig. 1.

### RNA isolation, cDNA preparation, and quantitative PCR

Total RNA was isolated from midgut and carcass samples using RNAeasy mini kit (Qiagen, Hilden, Germany) with a slight modification of adding 30 μl β-mercaptoethanol/ml RLT buffer.

First-strand cDNA was synthesised using QuantiTect reverse transcription kit (Qiagen, Hilden, Germany) according to the manufacturer’s instructions, followed by semi-quantitative PCR using gene-specific primers. Primer sequences of all genes analysed during this study are mentioned in Supplementary Table 1. The midgut expressing ABC transporter genes were further analysed by qPCR using SYBR green supermix in an iQ^TM^5 multicolor detection system (Bio-Rad, Hercules, CA, USA), where the mRNA for mosquito ribosomal protein subunit S6 gene was used as a normalisation control [37]. The following PCR cycle parameters were used: initial denaturation at 95 °C for 3 min, 35 cycles of 10 s at 95 °C, 40 s at 55 °C, and 1 min at 72 °C. The fluorescence readings were taken after each cycle. A final extension at 72 °C for 10 min was performed, followed by a melting curve analysis. Fold changes were calculated using the ΔΔCt method as described before [40]. Based on the expression analysis, the ABC transporter gene of Subfamily G (*AaeABCG3*) was shortlisted for gene silencing and further analysis.

### dsRNA synthesis of control and target genes

A 218-bp fragment of the lacZ gene (control) was amplified using the primers (5′ to 3′) F-GAGTCAGTGAGCGAGGAAGC and R-TATCCGCTCACAATTCCACA and cloned into the pCRII-TOPO vector [38]. To prepare dsRNA of *AaeABCG3* (target gene), a 240-bp fragment of the gene was cloned in pCRII-TOPO vector that already had a T7 promoter site at the M13F end. T7 promoter at the other end of the fragment was incorporated by amplifying the cloned insert using the following primers: M13F-GTAAAACGACGGCCAGT and T7-M13R-CTCGAGTAATACGACTCACTATAGGGCAGGAAACAGCTATGAC. PCR amplification was carried out at 95 °C for 5 min, followed by 40 cycles at 95 °C for 30 s, 55 °C for 30 s, 72 °C for 30 s, and a final extension at 72 °C for 10 min. Amplicons were purified from the gel using the QIAquick Gel Extraction Kit (Qiagen, Valencia, CA, USA). To synthesise dsRNA, these purified amplicons tailed with T7 promoter sequences were subjected to in vitro transcription using the MEGAscript kit (Ambion, Austin). The synthesised dsRNA was further purified using a Microcon YM-100 filter (Millipore, Germany) and finally concentrated to 3 µg/µl in DNase and RNase-free water.

### Effect of target gene silencing on midgut bacteria

One–two-day-old female *Ae. aegypti* mosquitoes were injected with 69 nl of 3 µg/µl dsRNA of *AaeABCG3* (207 ng/mosquito) into their thorax using a nanojector (Drummond, Broomall, PA, USA). Control mosquitoes were injected with LacZ dsRNA in the same manner. Four days after injection, the efficiency of mosquito *AaeABCG3* silencing was analysed using the expression primers (5′ to 3′) Fw – CCGGCTTCTTCGTCAACTTC and Rev – CAGAC CGATGAACAGGCAAA. After successful *AaeABCG3* silencing, samples were collected from both control and *AaeABCG3*-silenced mosquitoes (sugar-fed without any immune challenges) to assess the gene-silencing impact on endogenous midgut bacteria and immunity.

**Fig. 2:**
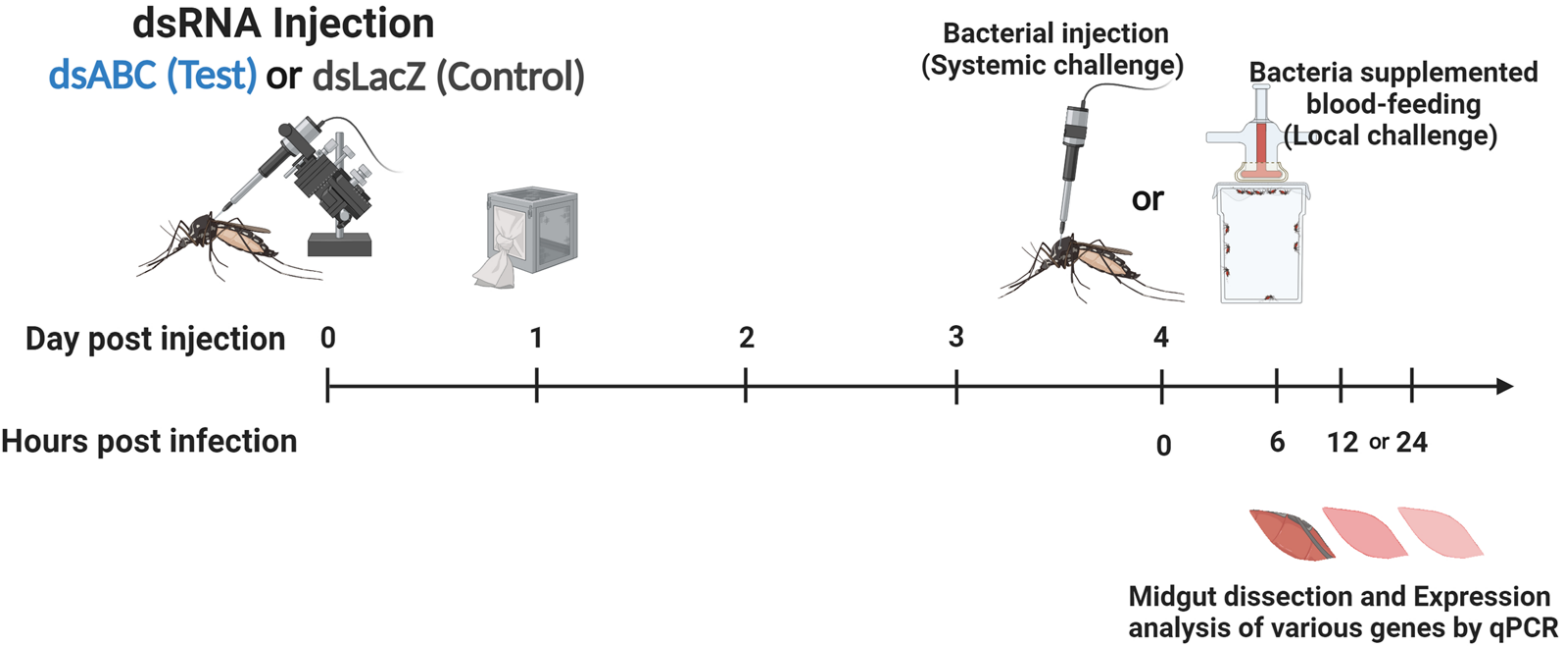
Schematics for RNAi-mediated functional characterisation of the target ABC transporter gene upon systemic and local immune challenges. (Created with BioRender.com)

Post-*AaeABCG3* silencing, mosquitoes were either allowed to feed on a mouse blood-supplemented bacterial mixture (local immune challenge) or a bacterial mixture was injected into the haemocoel (systemic immune challenge), as mentioned in Section “Systemic and local immune challenges to Ae. aegypti mosquito”. The impacts of *AaeABCG3* silencing on midgut bacteria and immunity were calculated by qPCR using 16S rRNA and immune gene-specific primers, respectively. Midgut samples were collected at early and late hours of bacterial immune challenges based on the results obtained from systemic and local immune challenges, as shown in Fig. 2.

RNA was isolated from these samples and used to synthesise first-strand cDNA, as mentioned in section “RNA isolation, cDNA preparation, and quantitative pcr". This was followed by qPCR for various ABC transporter genes using SYBR green supermix in an iQ^TM^5 multicolour detection system (Bio-Rad, Hercules, CA, USA). *Aedes aegypti* ribosomal protein subunit S6 gene was used as a normalisation control [37]. Fold changes were calculated using the ΔΔCt method as described before [40].

### Statistical analysis

All the data were expressed as mean ± standard deviation (SD). Statistical significance between the test and their controls was analysed using GraphPad Prism 8.0, a statistical software package (GraphPad Software, La Jolla, CA, USA). The type of test applied in the analysis is detailed in its respective figure legend. The data with a *p*-value < 0.05 were considered significant and indicated with asterisks (*p < 0.05; **p < 0.01; ***p < 0.001; ****p < 0.0001; ns = not significant).

## Results

### Identification of ABC transporter genes in *Ae. aegypti* midgut upon immune challenges

The organ-specific microbial-induced ABC transporter genes of *Ae. aegypti* were identified by challenging the mosquitoes with a mixture of gram-positive (*E. coli*) and gram-negative bacteria (*M. luteus*) using local and systemic routes (Fig. 1A). On monitoring the survival rate of mosquitoes for 7 days post immune challenges, we observed ≈100% survival in the case of local immune challenge, while the survival rate reduced to 50–60% during the systemic challenge.

**Fig. 3:**
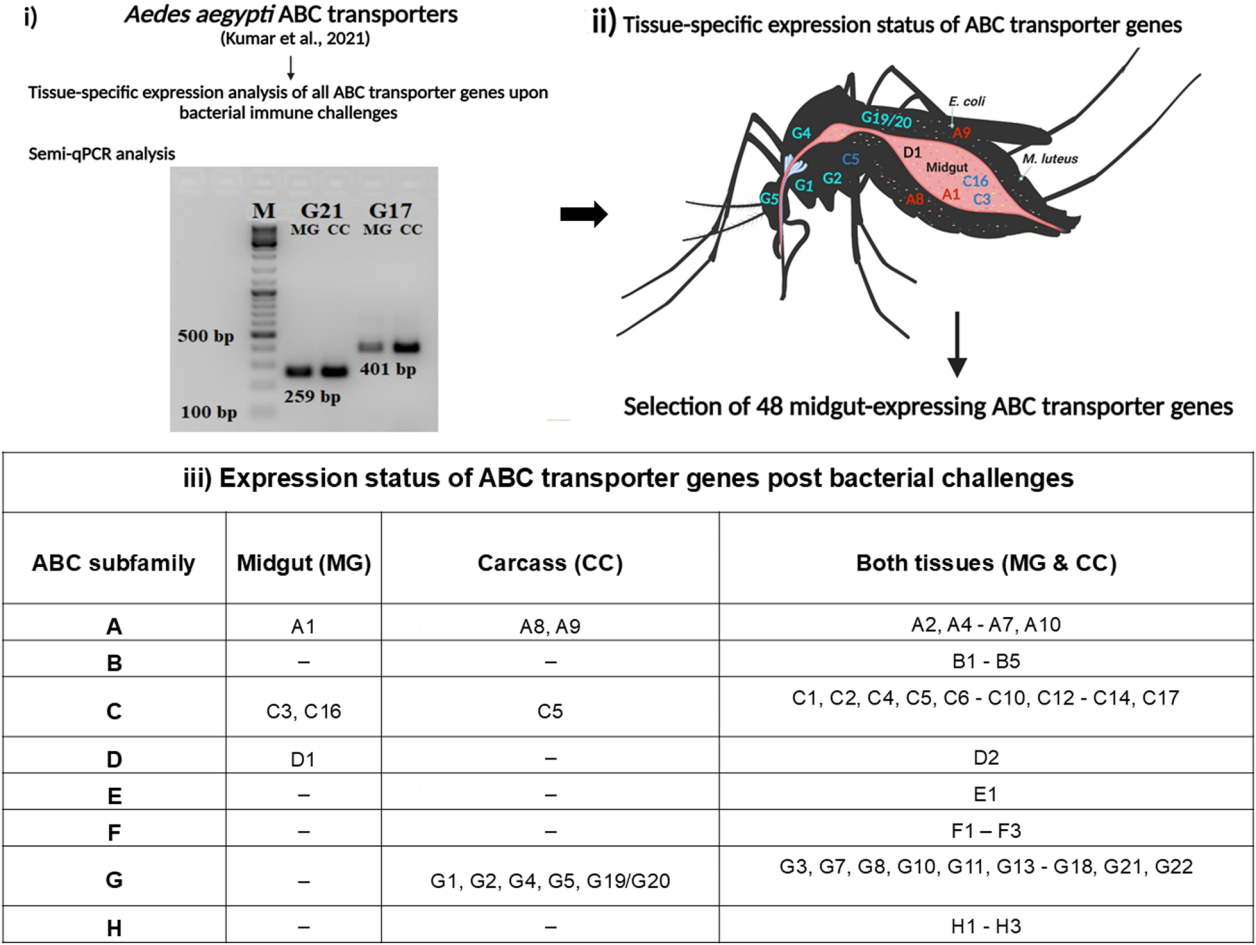
Tissue specific identification of ABC transporters upon bacterial immune challenges: **i** Identification and selection of midgut-expressing *Aedes aegypti* ABC transporter genes through semi-quantitative PCR post bacterial challenges. **ii** Expression profile of ABC transporter genes exclusive to the midgut or carcass (depicted in the mosquito cartoon). This figure was created with BioRender.com. **iii** Expression status of *Ae. aegypti* ABC transporter genes in the midgut, carcass or both tissues post bacterial challenges. M, marker; MG, midgut and CC, carcass

To analyse the expression of ABC transporters in the midgut (Mg) and carcass (Cc), we amplified all 59 ABC transporters [28] using semi-quantitative PCR (Fig. 3i). Overall, 48 ABC transporter genes were expressed in the immune-challenged midgut of *Ae. aegypti*, of which only a few were expressed exclusively in the midgut, as depicted in Fig. 3ii, iii.

### Systemic immune challenge induces transcriptional alteration of AMPs and ABC transporter genes in the midgut of *Ae. aegypti*

#### Live bacterial challenge (systemic immune challenge)

**Fig. 4:**
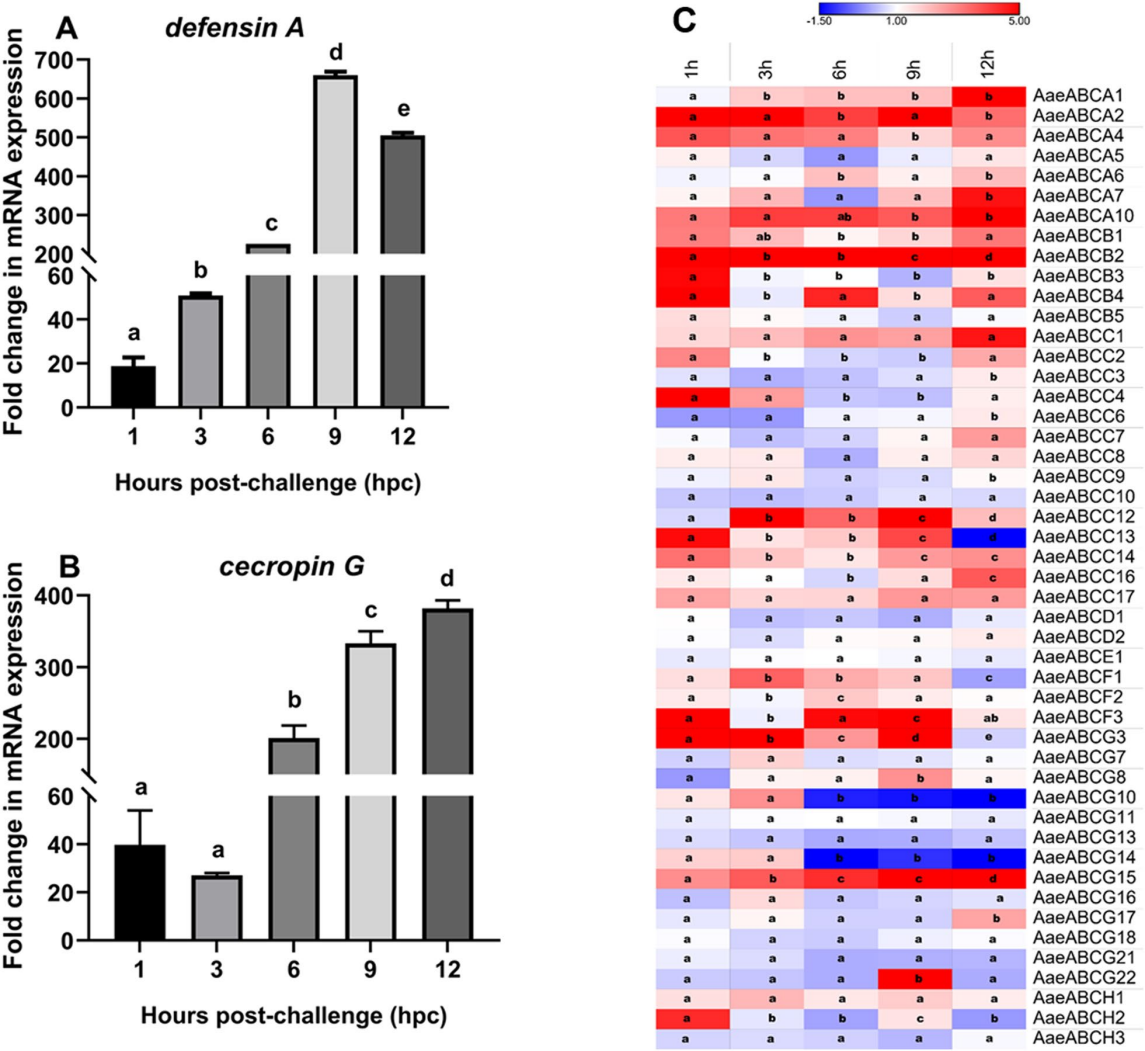
Expression analysis of AMPs and ABC transporter genes post-live bacterial (systemic) challenge. **A** Relative fold change expression of *defensin A*, **B** *cecropin G*, and **C** midgut-expressing *Aedes aegypti* ABC transporter genes by qPCR at various time points post-bacterial challenge. All samples were collected from three biological replicates (*n* = 10 per replicate) for each treatment (systemic immune challenged v/s sham-treated controls) at 1-, 3-, 6-, 9-, and 12 hpc. Statistical significance was analysed by one-way ANOVA with Tukey’s multiple comparison test for the differential gene expression using GraphPad Prism 8.0 (GraphPad Software, La Jolla, CA, USA). Significant differences among relative mRNA expression are represented with letters like “a, b, c”. The colour scheme indicates the log_2_ fold change in mRNA expression of ABC transporter genes

The systemic immune challenge with live bacteria exhibited an early induction of the mosquito midgut immunity where *defensin A* (Fig. 4A) and *cecropin G* (Fig. 4B) showed ~ 20- and 40-fold upregulation at 1-h post bacterial challenge (hpc), respectively. Furthermore, these immune genes showed an increase in expression until 12 hpc (Fig. 4A and B). Similar transcriptional alterations were also observed for a few other ABC transporter genes post-live bacterial challenge (systemic immune challenge) in *Ae. aegypti* midgut (Fig. 4C).

Most members of *Aae*ABCA and *Aae*ABCB subfamilies exhibited upregulation at various time points, where *AaeABCA2*, *AaeABCA4*, *AaeABCA10*, and *AaeABCB2* exhibited continuous upregulation. In addition, upregulation was also observed for *AaeABCG3* and *AaeABCG15* at various time points. In contrast, most members of *Aae*ABCD, *Aae*ABCG, and *Aae*ABCH subfamilies exhibited downregulation post-live bacterial challenge, where *AaeABCD1*, *AaeABCG11*, *AaeABCG13*, *AaeABCG18*, *AaeABCG21*, and *AaeABCH3* were observed to be downregulated throughout the infection. Basal to reduced expression was observed for the *Aae*ABCE subfamily, while *Aae*ABCF subfamily members exhibit upregulation during the early hours of live bacterial challenge. *Aae*ABCC subfamily members showed mixed responses where *AaeABCC3, AaeABCC6*, and *AaeABCC9* were downregulated at 6 hpc but exhibited upregulation at 12 hpc (Fig. 4C).

#### Heat-killed bacterial challenge (systemic immune challenge)

**Fig. 5:**
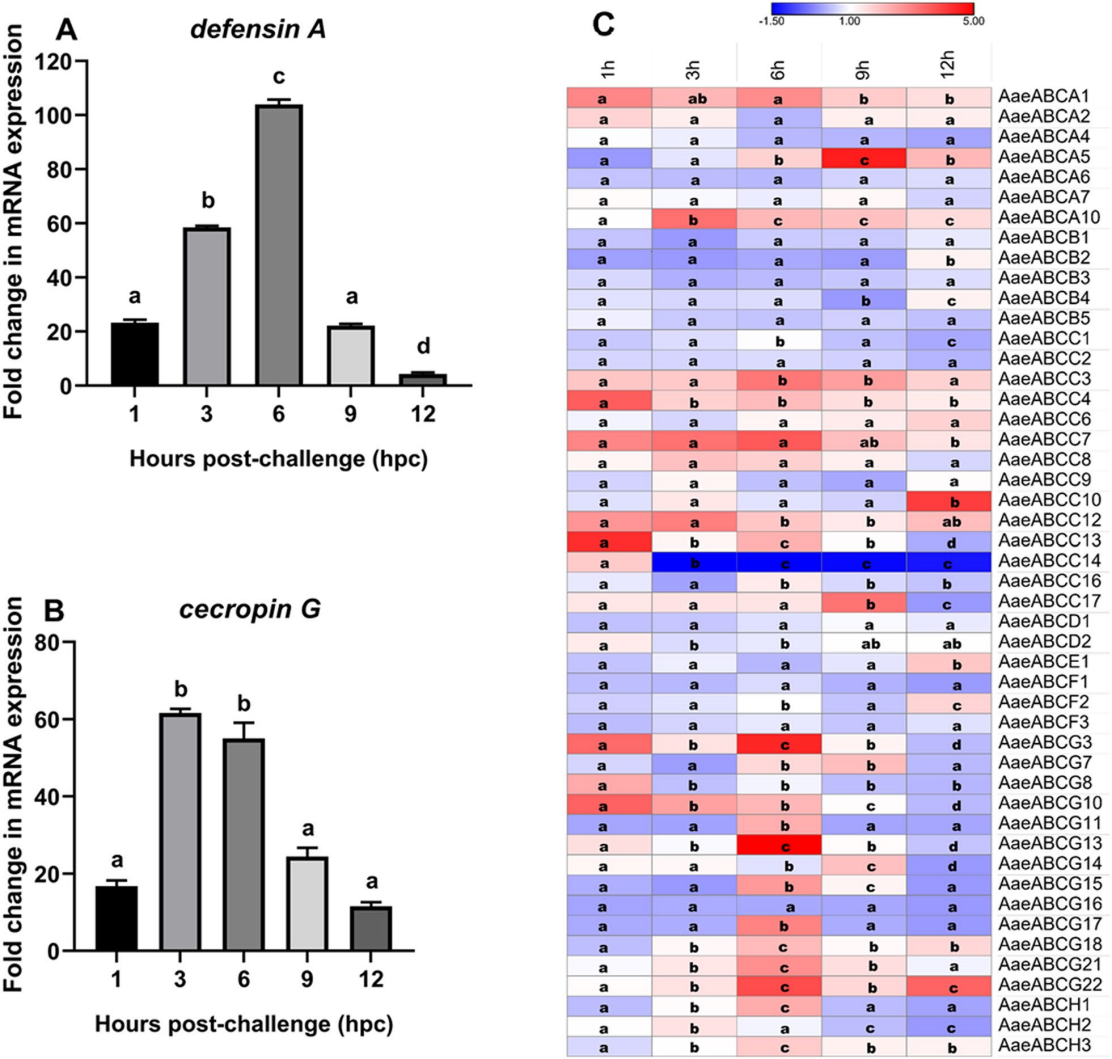
Expression analysis of AMPs and ABC transporter genes post-heat-killed bacterial (systemic) challenge to *Aedes aegypti*. **A** Relative fold change expression of *defensin A*, **B** *cecropin G*, and **C** midgut-expressing *Ae. aegypti* ABC transporter genes by qPCR at various time points post-nano-injection of the heat-killed bacterial mix. All samples were collected from three biological replicates (*n* = 10 per replicate) of each treatment (systemic immune challenged v/s control mosquitoes). Statistical significance was analysed by one-way ANOVA (GraphPad Software, La Jolla, CA, USA). Significant differences among relative mRNA levels are represented with letters like “a, b, c”. The colour scheme indicates the log_2_ fold change in mRNA expression of ABC transporter genes

Heat-killed bacterial challenge induced an early increase in the transcription of AMPs (Fig. 5A, B) and midgut-specific ABC transporter genes compared to the live bacterial challenge (Fig. 5C). The members of *Aae*ABCA and *Aae*ABCC subfamilies displayed varied expressions at various time points post-heat-killed bacterial challenges. *AaeABCA1* and *AaeABCA10* exhibited continuous upregulation, while *AaeABCA4* and *AaeABCA6* showed constant downregulation. In the *Aae*ABCC subfamily, *AaeABCC3*, *AaeABCC4*, *AaeABCC7* and *AaeABCC12* exhibited continuous upregulation, while *AaeABCC1*, *AaeABCC2*, and *AaeABCC14* were downregulated. Most members of *Aae*ABCB, *Aae*ABCD, *Aae*ABCE, and *Aae*ABCF subfamilies exhibited basal to reduced expression, suggesting their minimal role in mosquito immunity. In contrast, the AaeABCG and AaeABCH subfamilies members exhibited maximum upregulation at 6 hpc, slightly earlier than the live bacterial challenge (Fig. 5C).

Both live and heat-killed bacterial challenges (systemic immune challenges) induced transcriptional alteration of AMPs and ABC transporter genes in the midgut of *Ae. aegypti* (Figs. 4 and 5). While the live bacterial challenge exhibited a higher level of expression of AMPs and upregulation of many midgut-specific ABC transporter genes (Fig. 4), the heat-killed bacterial challenge showed just a notable early transcriptional increase of AMPs and upregulation of only a few midgut-specific ABC transporter genes, with maximum expression till 6 hpc, after which it started to decline (Fig. 5).

### Local immune challenges induce late transcriptional activation of mosquito immune and ABC transporter genes

**Fig. 6:**
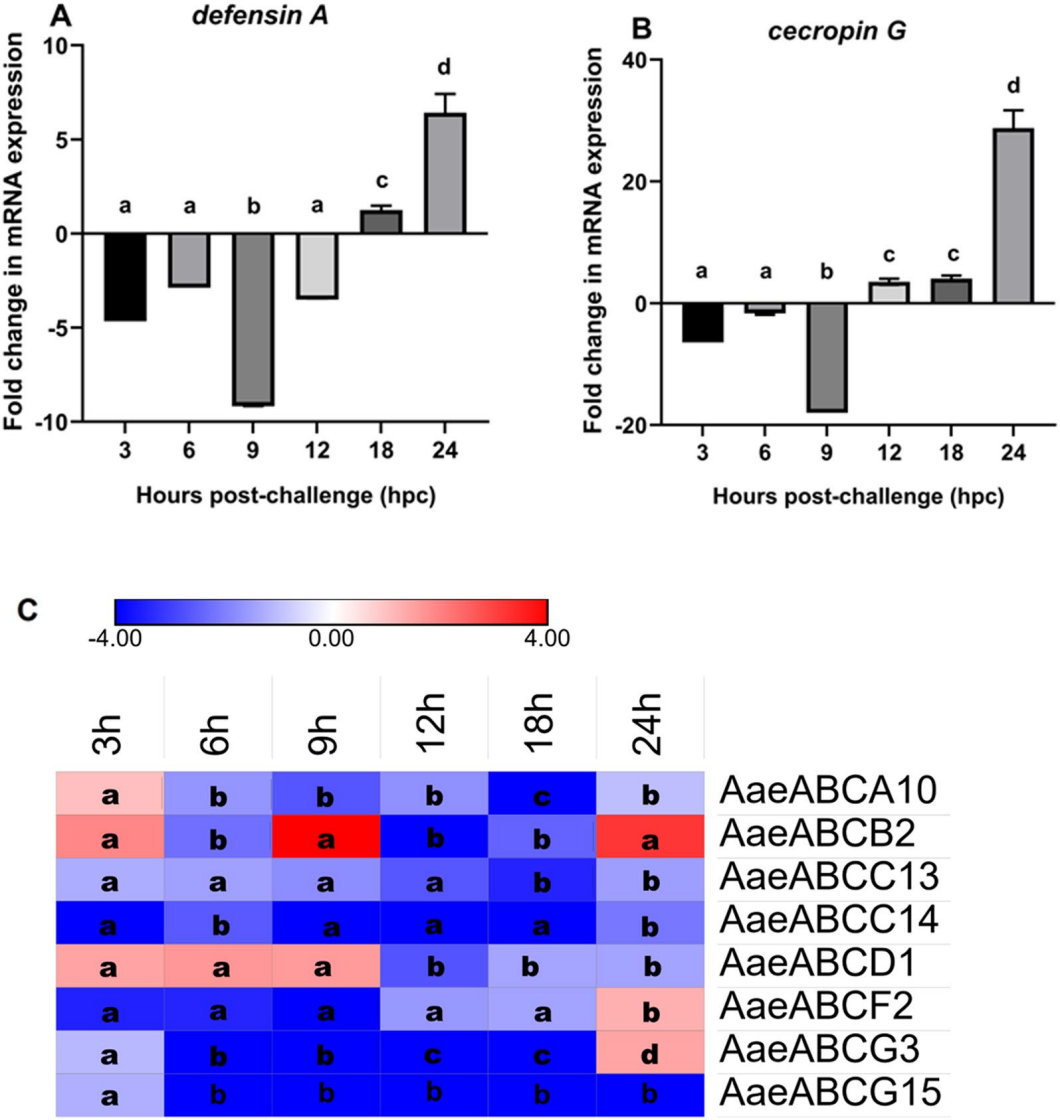
Expression profiling of antimicrobial peptides and ABC transporter genes upon local immune challenges. Relative fold change expression of **A** *defensin A*, **B** *cecropin G*, and **C** midgut-expressing *Aedes aegypti* ABC transporter genes by qPCR at various time points post-bacterial supplemented blood-feeding (local immune challenge). All samples were collected from three biological replicates (*n* = 10 per replicate) of each treatment (local immune-challenged test vs. control mosquitoes) at 3-, 6-, 9-, 18-, and 24 hpc. Statistical significance was analysed by one-way ANOVA with Tukey’s multiple comparison test for the differential gene expression using GraphPad Prism 8.0 (GraphPad Software, La Jolla, CA, USA). Significant differences among relative mRNA levels are represented with letters like “a, b, c”

Local immune challenges were introduced by artificial feeding of a gram-positive and gram-negative bacterial mixture to the female *Ae. aegypti* and the expression of AMPs and ABC transporters were analysed by qPCR (Fig. 6A, B). Only those genes that showed similar and contrasting expression profiles during systemic immune challenges (live and heat-killed bacteria challenges) were selected and investigated for their role in local immune response (Fig. 6).

A delayed immune response was observed, and the genes *defensin A* and *cecropin G* showed late induction with maximum expression at 24 hpc (Fig. 6A, B). Furthermore, we investigated the expression levels of the selected ABC transporter genes post-bacteria-supplemented blood-feeding (local immune challenge). Similar to *defensin A* and *cecropin G*, *AaeABCF2* and *AaeABCG3* also showed upregulation at 24 hpc. However, *AaeABCC13* and *AaeABCC14* showed downregulation throughout the local immune challenge (Fig. 6C). It is important to note that the systemic immune challenge of live bacteria exhibited constant upregulation of *AaeABCC14* (Fig. 4C). However, downregulation of this gene was observed post-heat-killed bacterial challenge (Fig. 5C).

Furthermore, to elucidate the role of ABC transporters in immunity, we analysed the expression profile of the ABC transporters in systemic and local immune challenges and selected a candidate gene, *AaeABCG3*, for further characterisation and to investigate its role in mosquito midgut immunity.

### Functional characterisation of *AaeABCG3* upon immune challenge through RNAi-mediated gene silencing

To characterise the *AaeABCG3* gene, the dsRNA of *AaeABCG3* (target) and LacZ (control) was injected into a group of mosquitoes (test and control group) as depicted in Fig. 2. The AMP (especially *defensin A*) induction represented two episodes, an early phase expression around 1 to 6 hpc and a late phase induction around 12 to 24 hpc. The gene-silenced and control midguts were collected at these two indicative time points to analyse the target and immune genes expression profile (as discussed in materials and methods) against bacterial immune challenges.

#### Silencing of *AaeABCG3* gene suppresses the native midgut bacteria by reactive species generation

**Fig. 7:**
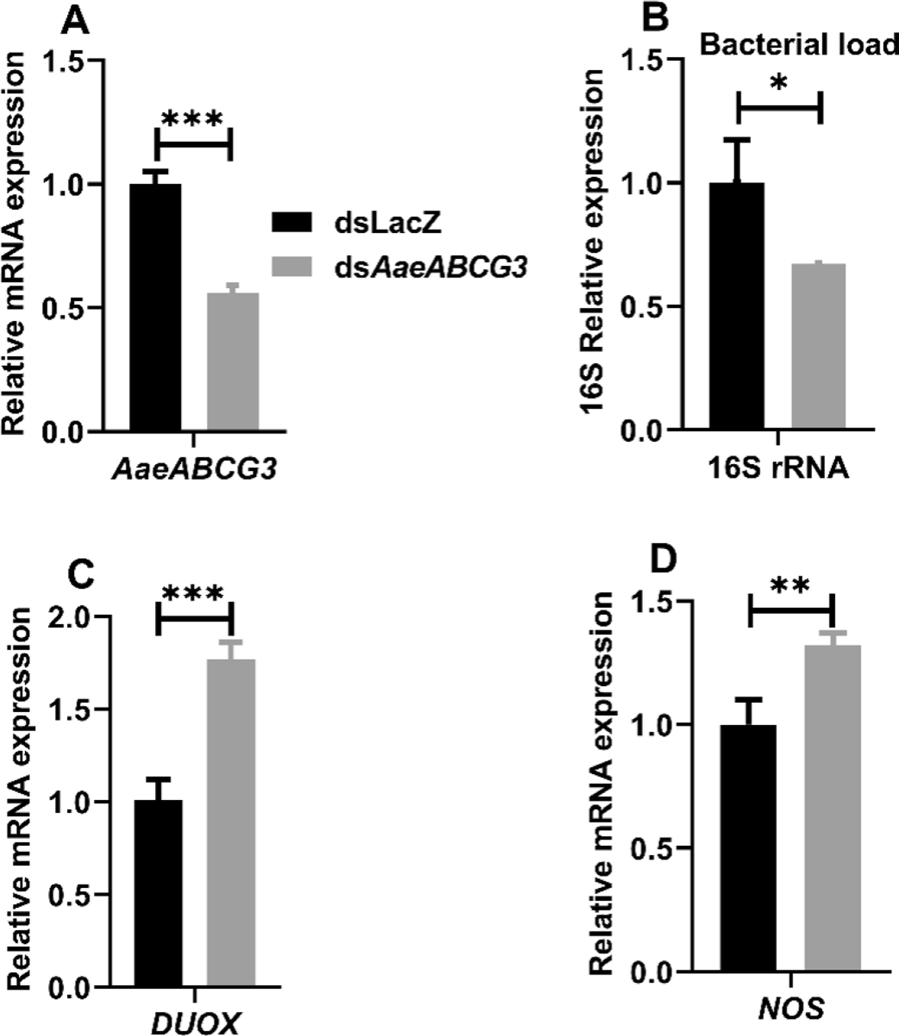
Effect of *AaeABCG3* gene silencing on endogenous bacteria and genes responsible for reactive species generation in midgut of sugar fed mosquitoes: Relative mRNA expression of **A** *AaeABCG3,* **B** 16S rRNA, **C** *DUOX*, and **D** *NOS* in *AaeABCG3*-silenced *Aedes aegypti* midguts (*dsAaeABCG3*, *n* = 10) compared to sham-treated controls (dsLacZ, *n* = 10) at 0 h of 4 days post-injection of dsRNA. Statistical significance was analysed by multiple t-tests with the Holm-Sidak method for the differential gene expression using GraphPad Prism 8.0 (GraphPad Software, La Jolla, CA, USA)

To illustrate the possible role of *AaeABCG3* in native midgut bacterial homeostasis, the expression of *AaeABCG3* gene in sugar-fed midguts of *AaeABCG3* gene-silenced mosquitoes was analysed against dsLacZ control mosquitoes. Expression analysis 4 days post-injection revealed a significant reduction in *AaeABCG3* mRNA levels in gene-silenced mosquito midguts compared to dsLacZ controls (Fig. 7A). Furthermore, we evaluated the gene-silencing effect on the growth of endogenous bacteria in the silenced midguts by analysing 16S rRNA levels (Fig. 7B). A significant (~ 1.5 fold) downregulation of 16S rRNA levels in the silenced midguts against sham-treated controls was observed.

These results prompted us to explore the immune regulations behind the endogenous bacterial suppression in *AaeABCG3*-silenced midguts. Therefore, we evaluated the expression of some well-known genes responsible for reactive oxygen/nitrogen species (ROS/RNS) like *DUOX* and NOS. We observed an increase of ≈1.8 and 1.3 fold in the *DUOX* and *NOS* mRNA levels respectively in the silenced sugar-fed midguts compared to the controls (Fig. 7C, D).

**Fig. 8:**
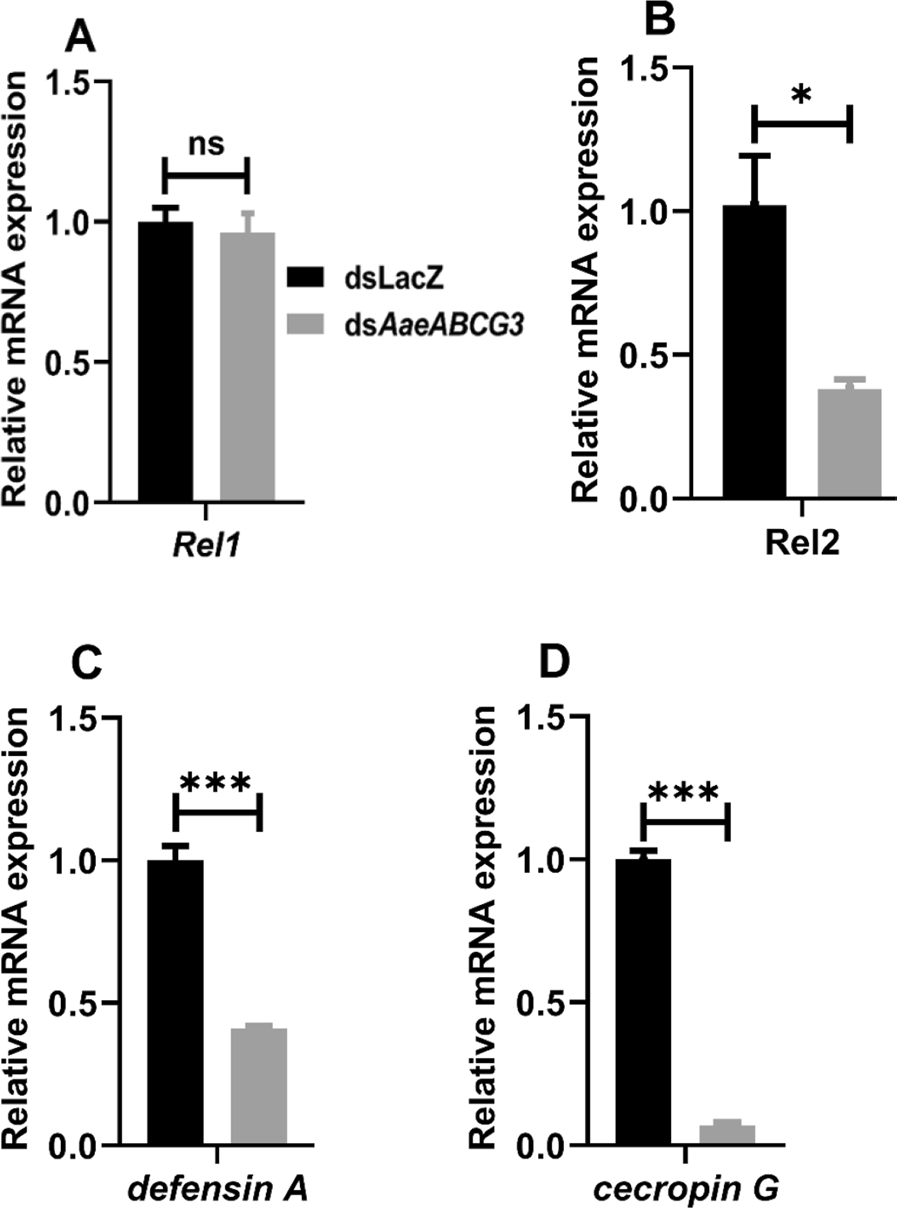
*AaeABCG3*-silenced mosquitoes exhibit suppressed immunity in sugar-fed midguts. Relative mRNA expression of **A** *Rel1,* **B** *Rel2*, **C** *defensin A*, and **D** *cecropin G* in *AaeABCG3*-silenced *Aedes aegypti* midguts (*dsAaeABCG3*, *n* = 10) compared to sham-treated controls (dsLacZ, *n* = 10) at 0 h of 4 days post-injection of dsRNA. Statistical significance was analysed by multiple t-tests with the Holm-Sidak method for the differential gene expression using GraphPad Prism 8.0 (GraphPad Software, La Jolla, CA, USA)

We further analysed the expression of *Rel1* and *Rel2* transcription factors of immune pathways and their immune effectors AMP genes, such as *defensin A* and *cecropin G*, in the silenced midguts against sham-treated controls (Fig. 8). We observed no change in the expression of *Rel1* (Fig. 8A). At the same time, *Rel2* exhibited significant downregulation in silenced midguts against sham-treated controls (Fig. 8B). A significant downregulation in *defensin A* and *cecropin G* expression (2.4 and 14.3 fold, respectively) was observed in *AaeABCG3*-silenced midguts compared to the controls (Fig. 8C, D).

#### Silencing of *AaeABCG3* gene modulates the midgut bacterial load during systemic bacterial challenge

**Fig. 9:**
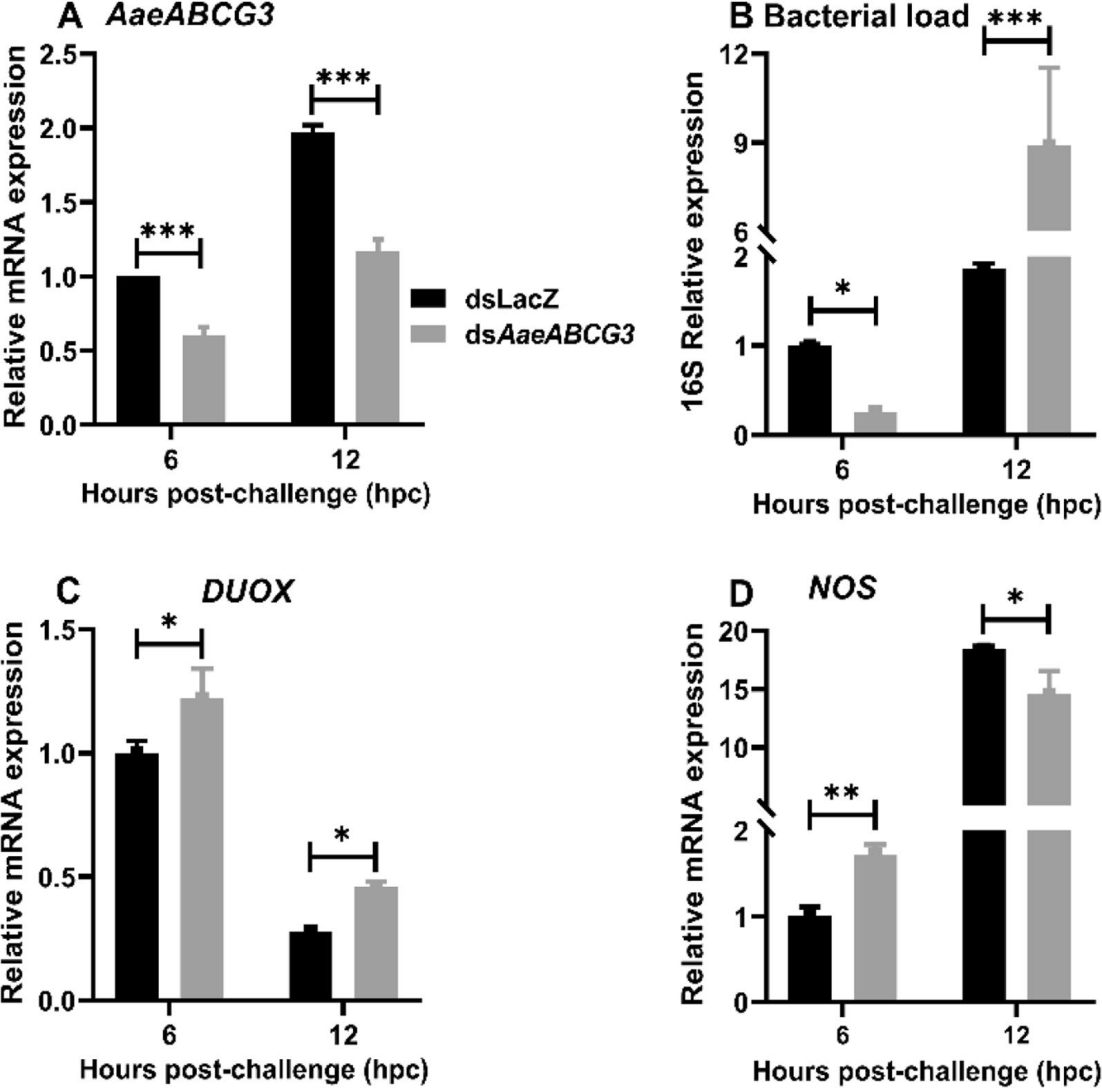
Effect of *AaeABCG3* gene silencing on midgut bacterial load and genes related to reactive species generation post-systemic bacterial challenge. Relative mRNA expression of **A** *AaeABCG3*, **B** 16S rRNA, **C** *DUOX*, and **D** *NOS* in *AaeABCG3*-silenced midguts compared to sham-treated controls post-systemic immune challenge. Statistical significance was analysed by multiple t-tests with the Holm-Sidak method for the differential gene expression using GraphPad Prism 8.0 (GraphPad Software, La Jolla, CA, USA)

To further understand the impact of *AaeABCG3* gene silencing on midgut immune regulation upon systemic bacterial challenge, the mosquitoes were injected with a bacterial mixture post-4-days of silencing, and the midgut samples were collected, as discussed in Fig. 2. The *AaeABCG3* expression in gene-silenced midguts was compared to sham-treated controls, revealing a significant silencing level of *AaeABCG3* (Fig. 9A).

Furthermore, we evaluated the impact of the systemic immune challenge on the midgut bacterial load at early (6 hpc) and late (12 hpc) time points by analysing 16S rRNA levels. Initially, the bacterial levels decreased by 3.85 fold; however, they increased to 4.76 fold later in the silenced midguts against sham-treated controls (Fig. 9B). We observed the induction of *DUOX* in the early and late hours of systemic bacterial challenge in *AaeABCG3*-silenced midguts compared to sham-treated controls (Fig. 9C).

We further analysed the expression of *NOS* in controls and *AaeABCG3*-silenced midguts upon the systemic bacterial challenge to understand the involvement of the JAK-STAT pathway in antibacterial immunity. We observed 1.7-fold induction for *NOS* in the early hours of systemic immune challenge (Fig. 9D), corroborating the reduction in midgut bacterial load in *AaeABCG3*-silenced midguts compared to sham-treated controls (Fig. 9B). However, a 1.26-fold reduction in the expression of *NOS* was observed in the late hours of the systemic immune challenge (Fig. 9D), which also corroborates the increase in midgut bacterial load in silenced midguts against sham-treated controls (Fig. 9B).

**Fig. 10:**
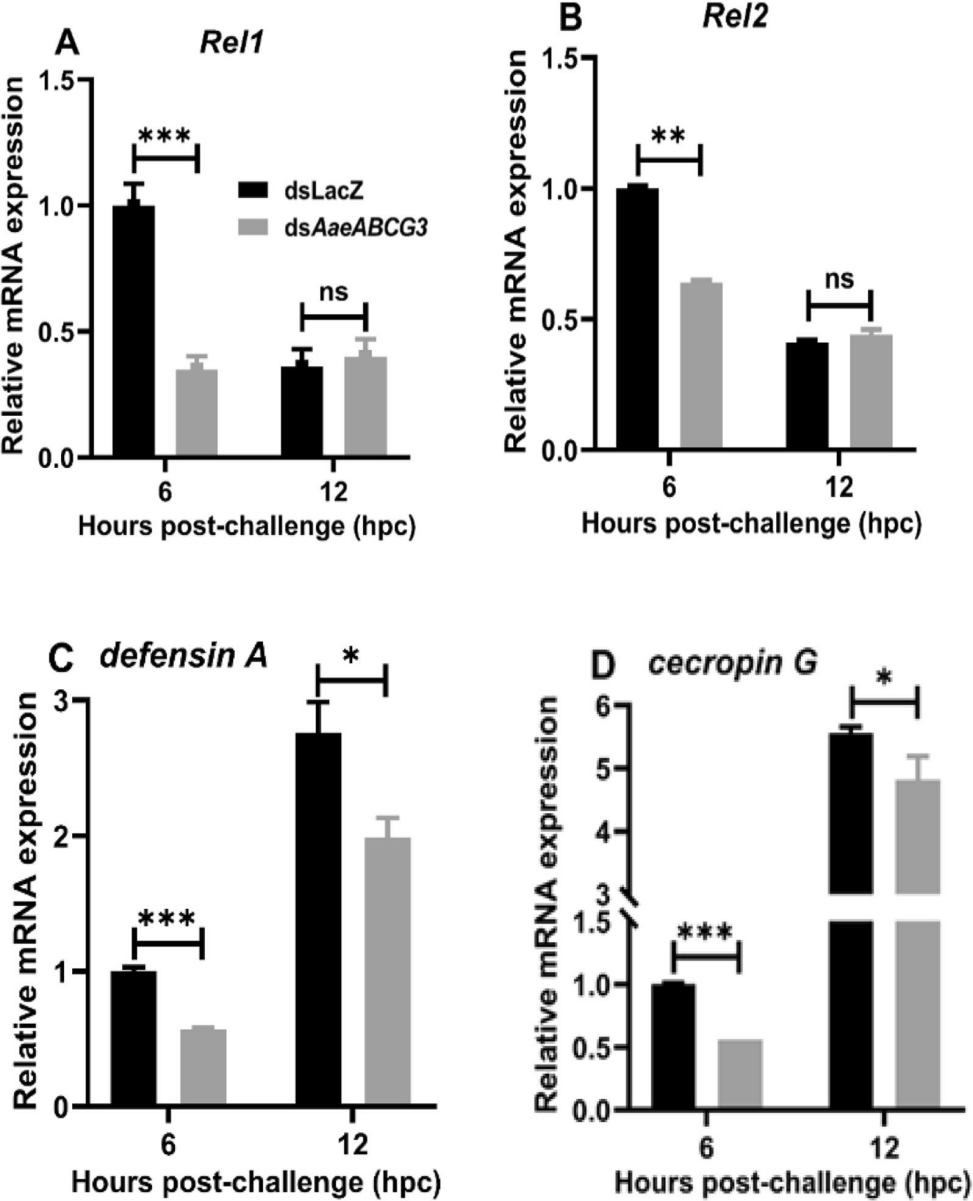
*AaeABCG3*-silenced mosquitoes exhibit suppressed immunity in systemic bacterial-challenged midguts. Relative mRNA expression of the transcription factors **A** *Rel1*, **B** *Rel2* and their immune effector genes, **C** *defensin A*, and **D** *cecropin G* in *AaeABCG3*-silenced midguts compared to sham-treated controls post-systemic immune challenge was assessed through qPCR using gene-specific primers. Statistical significance was analysed by multiple t-tests with the Holm-Sidak method for the differential gene expression using GraphPad Prism 8.0 (GraphPad Software, La Jolla, CA, USA)

To further understand the role of AMPs in *AaeABCG3*-silenced midgut upon systemic immune challenge, we analysed the expression profile of *defensin A* and *cecropin G* and their transcription factors *Rel1* and *Rel2* in the above samples. We observed significant downregulation of Rel 1 and Rel 2 in *AaeABCG3*-silenced midguts at early time points compared to their respective controls (Fig. 10A, B). In addition, the mRNA expression of the classical immune genes *defensin A* and *cecropin G* was significantly reduced in *AaeABCG3*-silenced midguts at both early and late time points compared to their respective controls (Fig. 10C, D), as observed previously for the sugar-fed *ABCG3*-silenced midguts (Fig. 8C, D).

#### Silencing of *AaeABCG3* suppresses the proliferation of bacteria in the midgut upon local immune challenge

**Fig. 11:**
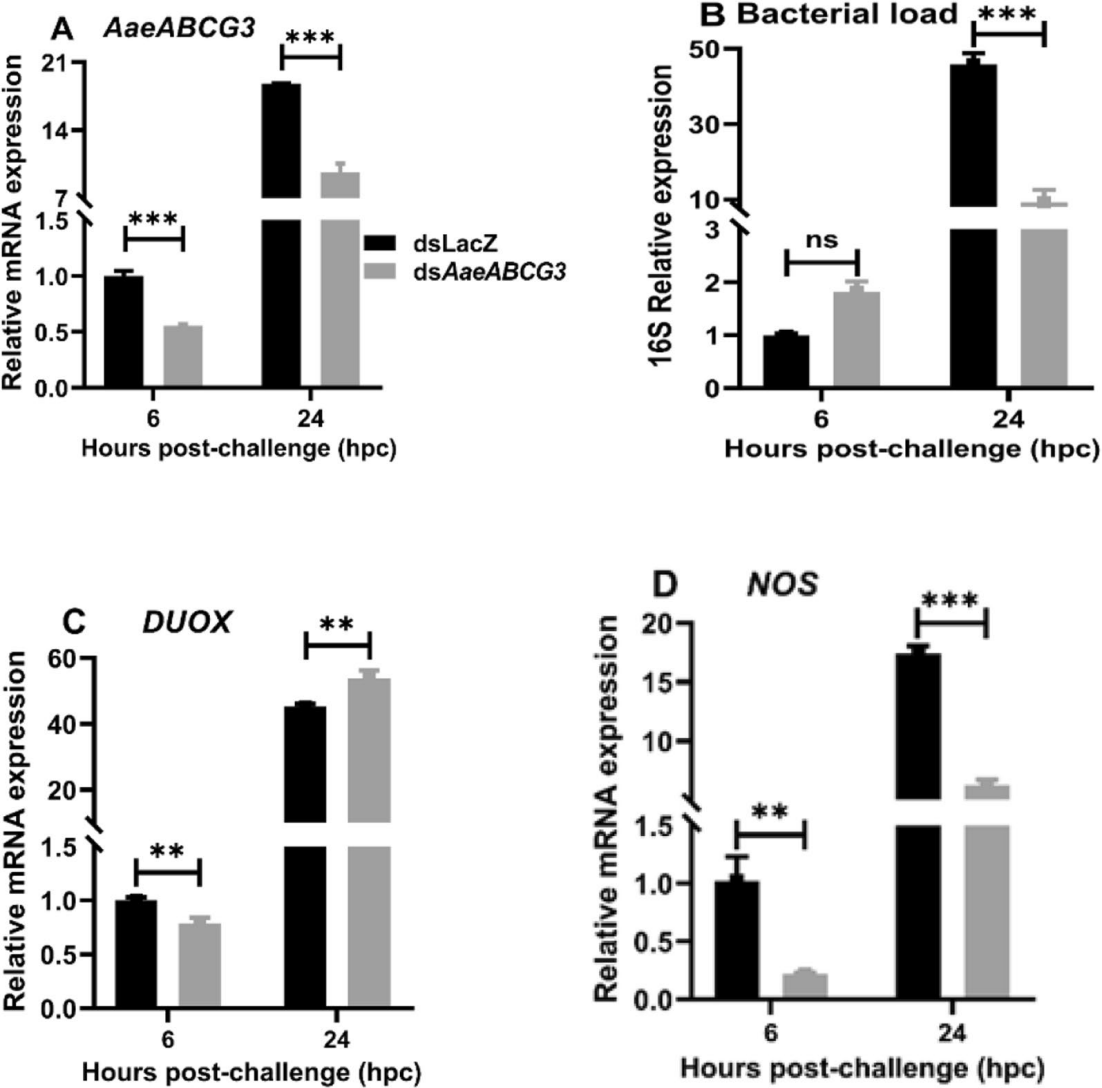
Effect of *AaeABCG3* gene silencing on midgut bacterial load and genes related to reactive species generation upon local immune challenge. Relative expression of **A** *AaeABCG3* mRNA, **B** 16S rRNA, **C** *DUOX*, and **D** *NOS* mRNA in local immune-challenged *AaeABCG3*-silenced midguts. Statistical significance was analysed by multiple t-tests with the Holm-Sidak method for the differential gene expression using GraphPad Prism 8.0 (GraphPad Software, La Jolla, CA, USA)

To understand the regulation of immunity upon local immune challenges in the *AaeABCG3*-silenced midguts, the mosquitoes were allowed to feed on the bacteria-supplemented blood meal post-4 days of dsRNA injection, as depicted in Fig. 2. The midgut samples were collected during the early (6 hpc) and late hours (24 hpc) of local immune challenges. We observed a significant reduction in mRNA levels of *AaeABCG3* in silenced midguts compared to the sham-treated controls (Fig. 11A). Furthermore, we evaluated the impact of the local immune challenge on the midgut bacteria by analysing 16S rRNA levels, which exhibited a 1.81-fold increase in midgut bacterial load at early stages of local immune challenges; however, at the late stage of local immune challenges, the bacterial load significantly decreased by 4.96 fold in the silenced midguts against sham-treated controls (Fig. 11B). Thus, the silencing of *AaeABCG3* reduced the overall proliferation of the bacteria in the midgut bolus, which might include endogenous and exogenous bacteria.

We further investigated the expression of the *DUOX* and *NOS* genes to understand the involvement of reactive species in antibacterial immunity in *AaeABCG3*-silenced midguts against controls upon local immune challenge. We observed a significant downregulation of the *DUOX* gene in the early hours. In contrast, this gene exhibits upregulation in the late hours of the local immune challenges against sham-treated controls (Fig. 11C). However, we observed a significant downregulation of the *NOS* gene at the early and late hours of the local immune challenges (4.64 and 2.78 fold, respectively) against sham-treated controls (Fig. 11D).

**Fig. 12:**
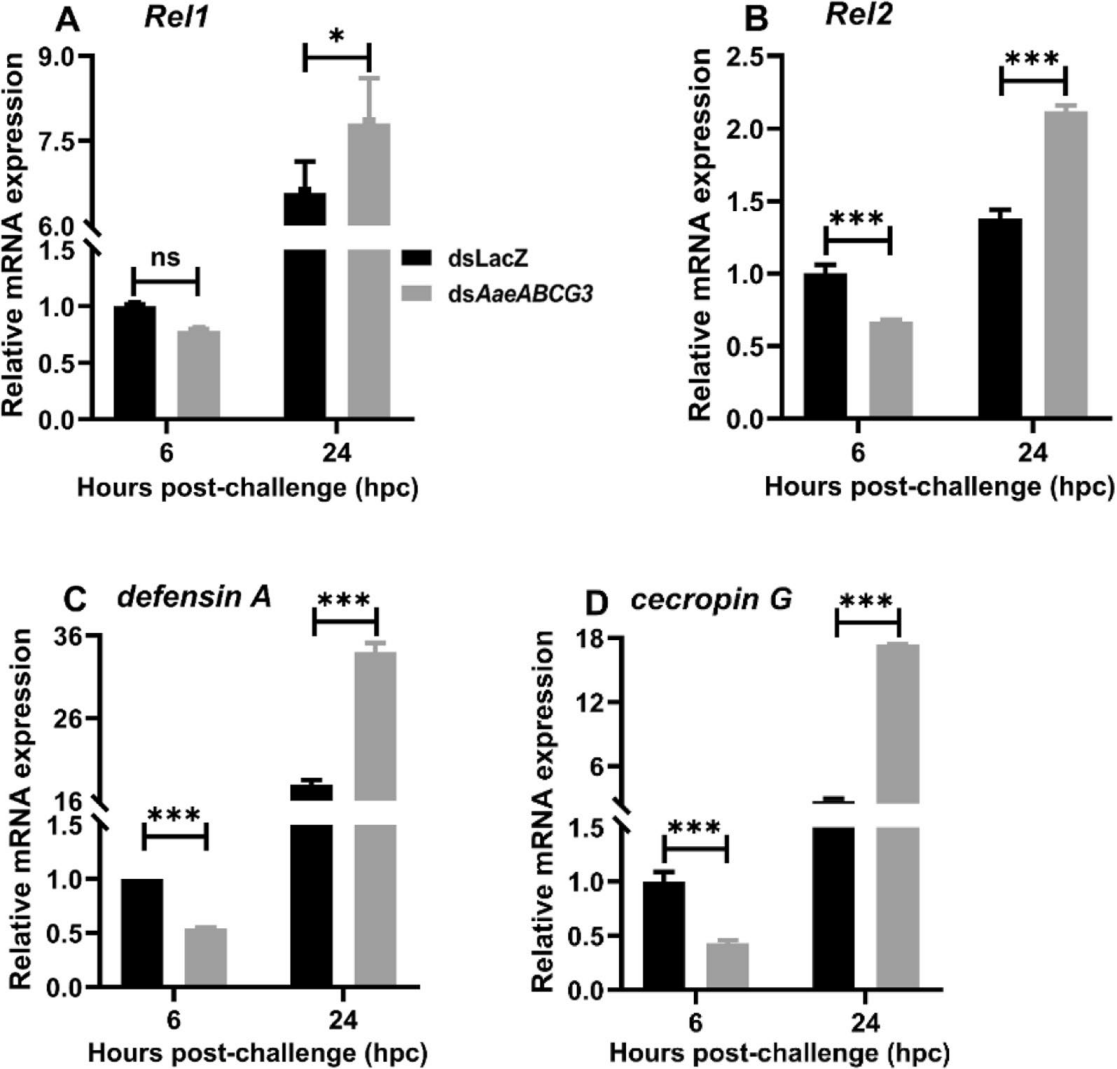
*AaeABCG3* gene-silenced mosquito exhibits elevated midgut immunity during the late hours of local bacterial challenge. Relative mRNA expression of **A** *Rel1*, **B** *Rel2*, **C** *defensin A*, and **D** *cecropin G* in *AaeABCG3*-silenced midguts compared to sham-treated controls post-bacterial supplemented feeding (local immune challenge). Statistical significance was analysed by multiple t-tests with the Holm-Sidak method for the differential gene expression using GraphPad Prism 8.0

Furthermore, when we analysed the expression of AMPs and their transcription factors, we observed significant upregulation of Rel1 and Rel2 in the late hours of local immune-challenged *AaeABCG3*-silenced midguts against their respective sham-treated controls (Fig. 12A, B). Both *defensin A* and *cecropin G* genes were significantly downregulated in the early hours of the local immune challenge, which might help the midgut bacterial propagation (Fig. 11B). However, at the late hours of the local immune challenge, *defensin A* and *cecropin G* were significantly upregulated (Figs. 12C, D), leading to the suppression of midgut bacteria, which is also evident by significant downregulation of 16S rRNA expression (Fig. 11B).

## Discussion

The mosquito midgut is essential for acquiring nutrition, immunity, and female fecundity due to the digestion of blood meal. It is an admirable system to understand the interaction of innate immunity, natural microbial habitats, and exogenous non-infectious or infectious meals. It is a critical organ that determines the vector competence for ingested pathogens [4, 41]. Most immune responses to various infections rely on activating immune signalling pathways by producing AMPs and reactive oxygen and nitrogen intermediates [1, 22, 42]. The midgut epithelial cells have been reported to be involved directly in innate immunity and can also perform inter-organ communication with the fat bodies and haemocytes [22]. Thus, to analyse the impact of ABC transporter on immunity, we initiated our experiments by identifying microbe-induced ABC transporter genes expressed in *Ae. aegypti* midgut post local and systemic bacterial challenges.

We identified expression for 48 *Ae. aegypti* ABC transporter genes following semi-quantitative PCR; however, for a few members of subfamilies *Aae*ABCA (*AaeABCA8* and *AaeABCA9*), *Aae*ABCC (*AaeABCC5*), and *Aae*ABCG (*AaeABCG1*, *AaeABCG2*, *AaeABCG4*, *AaeABCG5*, and *AaeABCG19*/*G20*), no expression was detected post-immune challenges. The literature survey and existing data analysis suggested that the undetected genes such as *AaeABCA8* and *AaeABCA9* might be involved in mosquito host-seeking behaviour or development as these were highly expressed in *Ae. aegypti* brain and sensory organs like antennae, legs, proboscis, and palps [43]. The uninduced ABC transporter genes included members of *Aae*ABCG subfamily that are related to eye pigment transportation, like white (*AaeABCG1*), scarlet (*AaeABCG2* and *AaeABCG4*), and brown (*AaeABCG5*). These genes are expressed during various developmental stages of mosquitoes, emphasising their exclusive role in insect eye pigmentation [28, 44, 45].

Furthermore, we investigated the transcriptional expression of microbial-induced midgut-expressing *Ae. aegypti* ABC transporter genes upon both systemic (intrathoracic microinjections) and local (infectious meal) immune challenges (Figs. 4–6). We observed early immune response to the systemic immune challenges, as reported previously [22], where both known AMPs exhibited early induction. In addition, we also observed early induction of some ABC transporters from subfamily *AaeABCA*, *AaeABCB*, and *AaeABCG.* During local immune challenges, our results exhibited downregulation of classical AMPs, *defensin A* and *cecropin G*, shortly after an infectious blood meal, which increased significantly at the later time point. This supports the recent report demonstrating the role of local immunity in the defence against ingested pathogens in mosquitoes [22]. Similar to the expression of AMPs, we also observed initial downregulation of *AaeABCF2* and *AaeABCG3*, which have increased lately, suggesting their possible involvement in a late local immune response (Fig. 6). All these observations relate to previous reports stating the importance of balancing immunity with tolerance to protect midgut microbiota from mosquito immunity [46].

Based on the observations made through systemic and local immune challenges and following the similar expression profile compared to classical immune gene *defensin*, we selected *AaeABCG3* gene from the pool of genes exhibiting modulation post-bacterial challenges for further analysis. RNAi-mediated gene silencing of *AaeABCG3* in the sugar-fed mosquitoes revealed significant downregulation of the 16S rRNA level, suggesting its potential role in maintaining endogenous bacterial homeostasis in mosquito midguts.

As per recent reports, the route of infection significantly impacts mosquito immunity against exogenous bacteria [47, 48]. Thus, we further explored *AaeABCG3* silencing’s impact on mosquito midgut immune regulation upon the exogenous bacterial immune challenge administered through the systemic and local routes and observed a significant decrease in expression of *defensin A* and *cecropin G* in *AaeABCG3* silenced midgut against sham-treated control mosquitoes upon both systemic and local (only early hpc) bacterial challenges, which suggested downregulation of the classical Toll and/or Imd immune pathways. However, the reduction in midgut bacterial load did not correlate with the downregulation of AMPs. In addition, the downregulation of AMPs in the gene-silenced mosquitoes was in contrast to the general systemic immune response where the AMPs showed an early response, suggesting that *AaeABCG3* silencing somehow suppressed the vector classical innate pathways during exogenous bacterial infection.

Further analysis revealed an overall corroboration in *DUOX* and *NOS* levels in *AaeABCG3* gene-silenced sugar-fed and systemically bacterial-challenged mosquitoes, with 16S rRNA expression (bacterial load), suggesting the possible involvement of JAK-STAT immune pathway in *AaeABCG3*-silenced mosquitoes. Our results parallel earlier reports, suggesting that the reactive species are continuously present in the midgut of sugar-fed mosquitoes [20]. It also suggests that blood feeding suppresses reactive species generation in the mosquito midgut [20]. This coincides with our results for local immune challenges where the downregulation of the *DUOX* (early hours) and *NOS* gene was observed in *AaeABCG3* gene-silenced mosquitoes against the sham-treated controls. However, an increase in the classical immune genes *defensin A* and *cecropin G* during late hours of local immune challenges in the gene-silenced mosquitoes compared to sham-treated controls suggests that the mosquito midgut immunity recuperates via the Toll or Imd pathways to maintain midgut bacterial homeostasis. These findings parallel previous reports, indicating that the midgut has an extraordinary ability to manage midgut microbiota homeostasis to avoid the deleterious effects of increased bacterial load on mosquitoes [4, 20, 38, 49–51]. Cumulatively, these findings support earlier reports suggesting that, in mosquitoes, blood-feeding induces formation of the peritrophic membrane, which acts as a barrier between ingested pathogens and midgut immunity [4, 52, 53]. However, when there is an increase in the bacterial load in late hours, the midgut displays an immune response by inducing expression of AMPs like *cecropin G* to maintain midgut homeostasis.

## Conclusions

This work reports molecular evidence for the connection between different immune stimulations and the transcriptional regulation of the midgut-specific ABC transporter genes. One of the ABC transporter genes, *AaeABCG3*, was funcionally characterized using RNAi-mediated gene silencing where it was seen to play a potential role in mosquito immunity. Notably, we have also found some other ABC transporter genes whose expression was significantly modulated during the systemic and local immune challenges. Further functional analyses of these ABC transporter genes will be desirable to elucidate their effective involvement in midgut immune activity. We believe that the work reported here provides useful indications in this direction.

## Supplementary Information

Additional file 1: Table S1A. The list of primers used for gene expression profiles of *Aedes aegypti* ABC transporters. Table S1B. The list of primers used for gene expression profiles of *Aedes aegypti* immune genes.

## Data Availability

The data used for this study is already available in the main text.
