# Supplementary material for: Midgut immune profiling and functional characterization of Aedes aegypti ABC transporter gene(s) using systemic and local bacterial challenges
Source: Parasit Vectors. 2025 Jan 31;18:34. doi: 10.1186/s13071-025-06658-6 (PMC11786363; doi:10.1186/s13071-025-06658-6)
Supplement: Supplementary file 1 — Additional file 1: Table S1A. The list of primers used for gene expression profiles of Aedes aegypti ABC transporters. Table S1B. The list of primers used for gene expression profiles of Aedes aegypti immune genes. [file 13071_2025_6658_MOESM1_ESM.docx]

**Supplementary Table 1A:** Details of primer sequences of *Aedes aegypti* ABC transporter genes used for this study are provided below. **All of these primers were designed and synthesized during this study.**

| **Gene Name (VectorBase ID)** | **Forward primer sequences (5′ – 3′)** | **Reverse primer sequences (5′ – 3′)** |
| --- | --- | --- |
| ***AaeABCA1*** (AAEL012698) | ACATCTGCTCACGGGAAAAC | CGTTCATGTCCTCCTTCGTT |
| ***AaeABCA2* (*A2.1* & *A2.2*)** (AAEL012702 & AAEL012700) | CGGACAAAAGTTCCTCCTGC | GACTCCATGGTTTGAGCGTC |
| ***AaeABCA4*** (AAEL001938) | TGGGGTGTGATATGGCTGTT | ACCACTGCATTCCTTCGGTA |
| ***AaeABCA5*** (AAEL008386) | CGGACAATTATCGGACGCTC | TGGCTCGATCTACAGCACTT |
| ***AaeABCA6*** (AAEL008384) | CAGAGGATGGATTGCGATGC | TGTCAACGGTACAGTGTGGA |
| ***AaeABCA7* (*A7.1* & *A7.2*)** (AAEL017572 & AAEL014699 ) | GAATGACTCCGCGAACATCC | TGGGCTCGTCAAGAATCAGT |
| ***AaeABCA8*** (AAEL021738) | ATCTTCGATCACACCTCCCG | GCACGTCGAACACAGGATAC |
| ***AaeABCA9*** (AAEL018040) | GATGTTCTTTGCGGGGTTGA | TGCAGTAGTTCCCTCCGTTT |
| ***AaeABCA10*** (AAEL012701) | CGGACTTGCTTCGAGATTGG | ACCACTGTTGAAACCATCGC |
| ***AaeABCB1*** (AAEL008134) | CTTCGGGTGCAGGTAAGAGT | GGTTTCGTACTGATCGGGGA |
| ***AaeABCB2*** (AAEL010379) | TTGAGAAAGGAGTGGTCGCT | ATGCTCCTACAACCATGGCT |
| ***AaeABCB3*** (AAEL022941) | AGAGTCAAGCCCAATCGGAA | CGAGAACCAGCGTCGTTTAG |
| ***AaeABCB4*** (AAEL006717) | CAGGAGGAGAAAAGCAACGG | TTCCTTGGGACCGAGCTTAG |
| ***AaeABCB5*** (AAEL000434) | GGGCCAATTTGACACCTGTT | CACCTGCCAGCAACATACAA |
| ***AaeABCC1*** (AAEL005918) | ACCATTAGAGTCCGGGCAAA | CCTCCCGACAGTGAGATACC |
| ***AaeABCC2*** (AAEL025460) | GTGATCTTCATCGTGACGCC | CGTGTTCGCTAGGATTGCAA |
| ***AaeABCC3*** (AAEL005929) | ATCGTACATTCGCGGGATCT | AGTAGGGCACACTTTCACCA |
| ***AaeABC4* (*C4.1* & *C4.2*)** (AAEL012395 & AAEL019847) | CTCATTTCGGTGCTGCGAAT | AATTCACCACACGCTTCGAC |
| ***AaeABCC5*** (AAEL023958) | GATCTCGATACGGCGCATTG | GGCATCCTTGTTCCAACTGG |
| ***AaeABCC6*** (AAEL027539) | GATTGAGACGCGACGAGATC | CCTCAAACACCCAAGGCTTC |
| ***AaeABCC7*** (AAEL018267) | ATGACCATCTTCTTCCCGCA | AGGTCCAATAACAGCCACCA |
| ***AaeABCC8*** (AAEL005045) | GAACGCGACTTCTGGTAACC | TGCTGTTTCGAGACTCCTGT |
| ***AaeABCC9*** (AAEL005026) | CTTCCCAGACGAACAGCCTA | CCTTGACAATGACCGGCAAA |
| ***AaeABCC10*** (AAEL005043) | AAAGCCCTCGAACTGTCTCA | ACAATACCCGCATTCTTCGC |
| ***AaeABCC12*** (AAEL020303) | TCAGCTTCCACTCCTTCTGG | GACAGATAGCCGACCTCCTC |
| ***AaeABCC13*** (AAEL023524) | TTGCTCTCGATTACCTGGGG | ACACGATGTCTCCCTTCTGG |
| ***AaeABCC14*** (AAEL004743) | TTTTGCCACCGTAATCCTGC | TCCGACCATCTCCAAACGAA |
| ***AaeABCC16*** (AAEL017209) | ACACACTGCAATACTCGCTG | TAGCCATCTGTTTCCGACGT |
| ***AaeABCC17*** (AAEL015644) | TTACGCGCTACACAAACCAC | TGAGTCGCTTAGAACGTCGA |
| ***AaeABCD1*** (AAEL010047) | AGAAGACGGTGGTATCTGCG | CATACACGGTTTACCCTGCG |
| ***AaeABCD2*** (AAEL002913) | CATTACGCTGTTCACCGTGT | GTTTCCTGCACCTCATCCTG |
| ***AaeABCE1*** (AAEL010059) | AGGAGAACTGCAGCGTTTTG | TCATCCGGCTCCAGATTACC |
| ***AaeABCF1*** (AAEL001101) | AGAAGGCAGTAGTCGATCCG | ACCTTCACCTCTTGCTCCTC |
| ***AaeABCF2*** (AAEL010977) | CCAGTGCGTTATGGAAGTCG | GCAAGCGTCCAAATCGAGAT |
| ***AaeABCF3*** (AAEL010359) | GCGGATCTGTTACTGGCTTG | AGCAGATCGGGTTTGGAGAA |
| ***AaeABCG1*** (AAEL016999) | CTGGAAGTGTTGTGTGGTGG | TTGCACTTTTGTTCGTTGCG |
| ***AaeABCG2*** (AAEL021570) | ATCCTCATGATCACCTCCGG | GGTGTGACTCGTCGAAGCTA |
| ***AaeABCG3*** (AAEL008138) | CCACCATTCCGATCATCCTG | TCGCATCGATCCAGTACTCG |
| ***AaeABCG4*** (AAEL003703) | TCGTGGATGATGTTTGCCAG | TCTTTGAAGCTCGCCTATGC |
| ***AaeABCG5*** (AAEL017188) | AGTGCGGGAACATCTGTCAT | AACTATCTAGGCCCGTGGTC |
| ***AaeABCG7*** (AAEL008672) | GCTGTACACTCTTGCCGATG | AAGTCCAACTCACCCCGAAT |
| ***AaeABCG8*** (AAEL019463) | TCGTTTCTCAAGTACGCCCT | CAGCTCTAGTAGACGTGGGG |
| ***AaeABCG10*** (AAEL027367) | AAGGACATGGACATCGAGCA | ACCAAAGTACAAACAGCGTCC |
| ***AaeABCG11*** (AAEL008635) | GAGTTACACTGCGCGATCTG | TTGAGGGGTGGGAATTGGAA |
| ***AaeABCG13*** (AAEL022734) | GAAACGATCGCCATGCAAAC | ACGATACAGGTCAGCAGGAG |
| ***AaeABCG14*** (AAEL027424) | CTTGCGTGCTGTATCCACTC | GAACAGTTCCACTTGCCAGG |
| ***AaeABCG15*** (AAEL019641) | GGTATGACGCCCCAGAACTA | CTACTGCCTCTTTCCCAGCT |
| ***AaeABCG16*** (AAEL008625) | CAAAGGGGTGGTGAAAGGTG | CCTGGGAGATGTAGACGCAT |
| ***AaeABCG17*** (AAEL008628) | AACGATGACTTACGCGGTTG | GCGTTGGAACCATACCTTCG |
| ***AaeABCG18*** (AAEL008632) | GAAGTGGCGAGTTTGAAGCA | CCACCTTCCAGAACACAACG |
| ***AaeABCG19/G20*** (AAEL026976) | GCTGCTGAAGAACATCTCCG | GCACGTCCTGAGTGTTGTAC |
| ***AaeABCG21*** (AAEL008624) | CTGCACTGAACCGGACAAAA | GCCGACGATGATTCCGAAAA |
| ***AaeABCG22*** (AAEL027686) | AGGGTCTTGCGTATTCCAGG | GGTTCCTCTTCCACTGCTGA |
| ***AaeABCH1*** (AAEL005491) | AAGAACCCGAACGTCATCCT | ATCGGTTTTCTCGTCCACCT |
| ***AaeABCH2*** (AAEL018334) | GTTCACATTACCAAGGCCGG | CGCTCTCCTGACTCACGTAA |
| ***AaeABCH3*** (AAEL014428) | ACTTTATGGCAACCGGAAGC | GATACCAACAGCACCGTAGC |

**Supplementary Table 1B:** Details of primer sequences for various genes of *Aedes aegypti* used for this study are provided below.

| **Gene Name**  **(VectorBase ID)** | **Forward primer sequences (5′ – 3′)** | **Reverse primer sequences (5′ – 3′)** | **References** |
| --- | --- | --- | --- |
| *Rel1*  (AAEL007696) | GACTCGTCGGAGCTGAAATC | CGGTTTGTTCAGGTTGTTGA | Barletta et al., 2017 |
| *Rel2*  (AAEL007624) | TCTGTCGGCAGATGAAGTGA | GCACTGGAATGGAGAATCAAA |  |
| *~~STAT~~*  ~~(AAEL009692)~~ | ~~CACACAAAAAGGACGAAGCA~~ | ~~TCCAGTTCCCCTAAAGCTCA~~ |  |
| *defensin A*  (AAEL003841)  or  *defensin C*  (AAEL003832) | GCGACCTGCGATCTGCTG | TCAATTTCGACAGACGCAGACCTT | Bartholomay  et al., 2004 |
| *cecropin G*  (AAEL015515) | TCACAAAGTTATTTCTCCTGATCG | GCTTTAGCCCCAGCTACAAC | Xi et al., 2008 |
| *NOS*  (AAEL009745) | CGCGACGAAAAAGAAGAGAT | GACATCACCGCAGACGTAGA | Coggins et al., 2012 |
| *DUOX*  (AAEL007563) | GGATTTGTGCCCATCCTATG | AACCGTGTAGATCGCTGCTT | Talyuli et al., 2023 |
| *HPX1* (AAEL006014) | TCCTGTGCATCCTGACTGAG | CGTTGTCGCAGAAGATACGA |  |
| RPS6 (AAEL000032) | CGTCGTCAGGAACGTATCCG | TCTTGGCAGCCTTAGCAGC | Molina-Cruz et al., 2005 |
| 16S rRNA  (Bacteria quantification) | \| TCCTACGGGAGGCAGCAGT \| \| --- \| | GGACTACCAGGGTATCTAATCCTGTT | Kumar et al., 2010 |
